# The Interplay Between Muscular Activity and Pattern Recognition of Electro-Stimulated Haptic Cues During Normal Walking: A Pilot Study

**DOI:** 10.3390/bioengineering11121248

**Published:** 2024-12-09

**Authors:** Yoosun Kim, Sejun Park, Seungtae Yang, Alireza Nasirzadeh, Giuk Lee

**Affiliations:** 1 School of Mechanical Engineering, Chung-Ang University, 84 Heukseok-Ro, Dongjak District, Seoul 06974, Republic of Korea; rladbtjs0930@cau.ac.kr (Y.K.); pmjk22673@cau.ac.kr (S.P.); 2 HUROTICS Inc., Heukseok-Ro, 25, Dongjak District, Seoul 06974, Republic of Korea; 99hilton@hurotics.com

**Keywords:** electrical stimulation, haptic cue, electromyography, pattern recognition, walking

## Abstract

This pilot study explored how muscle activation influences the pattern recognition of tactile cues delivered using electrical stimulation (ES) during each 10% window interval of the normal walking gait cycle (GC). Three healthy adults participated in the experiment. After identifying the appropriate threshold, ES as the haptic cue was applied to the gastrocnemius lateralis (GL) and biceps brachii (BB) of participants walking on a treadmill. Findings revealed variable recognition patterns across participants, with the BB showing more variability during walking due to its minimal activity compared to the actively engaged GL. Dynamic time warping (DTW) was used to assess the similarity between muscle activation and electro-stimulated haptic perception. The DTW distance between electromyography (EMG) signals and muscle recognition patterns was significantly smaller for the GL (4.87 ± 0.21, mean ± SD) than the BB (8.65 ± 1.36, mean ± SD), showing a 78.6% relative difference, indicating that higher muscle activation was generally associated with more consistent haptic perception. However, individual differences and variations in recognition patterns were observed, suggesting personal variability influenced the perception outcomes. The study underscores the complexity of human neuromuscular responses to artificial sensory stimuli and suggests a potential link between muscle activity and haptic perception.

## 1. Introduction

With its ability to convey tactile information through physical stimulation, haptic cue technology is revolutionizing numerous fields, including healthcare, rehabilitation, and human–computer interaction. Within this realm, electro-stimulated haptic feedback offers unique possibilities, directly activating muscles to generate sensations without relying on external mechanical interfaces [1]. Particularly in walking, the accurate recognition of haptic patterns becomes crucial for applications like navigation assistance, gait parameter feedback, and balance management in individuals with mobility impairments [2,3]. However, understanding how the neuromuscular system interprets and processes these artificial signals remains a crucial challenge.

Recent research reveals a captivating interplay between muscle activity and the perception of electro-stimulated haptic signals during walking. On the one hand, higher muscle activation, potentially reflecting focused attention and engagement of proprioception, might lead to a more accurate recognition of haptic patterns [4,5]. This intriguing link could be attributed to two potential mechanisms: firstly, the muscle activation might enhance brain sensory processing, making the stimulated sensations stand out and easier to discern. Secondly, a closed-loop system could be at play, where successful recognition of the haptic pattern triggers further muscle activity, amplifying and clarifying the sensation in subsequent stimulation cycles [6].

However, the influence is not unidirectional. That is, successfully recognizing haptic signals can also shape muscle activity patterns. Accurately interpreting signals might lead to calmer muscle responses, reflecting reduced uncertainty and anxiety associated with deciphering the sensations [7]. Additionally, depending on the specific context, like navigating an obstacle, accurate recognition could trigger specific and targeted muscle responses, resulting in distinct electromyography (EMG) patterns compared to situations characterized by inaccurate recognition [8]. Furthermore, over time, users might be able to modulate their muscle activity based on the haptic feedback they receive, essentially learning to optimize recognition through this experience-driven adaptation [9].

Despite the compelling possibilities, the research results to date are inconclusive. While some studies unveil positive correlations between EMG activity and haptic recognition accuracy [10,11,12], others show no significant connection [11,13]. This complexity likely arises from many factors, including individual differences in neuromuscular responses, variations in the type of stimulation employed, the specific demands of the walking task, and other yet-to-be-unraveled influences. Delving deeper into this intricate dance between muscle activity and haptic perception holds immense potential for optimizing haptic technology, particularly in the context of gait interventions, rehabilitation protocols, and assistive technologies for individuals with mobility impairments.

As the relationship between EMG activity and electro-stimulated haptic perception during walking is unclear, further investigation is crucial to understand it fully. In this study, we hypothesized that higher muscle activity would result in more accurate electro-stimulated haptic perception during normal walking. To test this idea, we provided electro-stimulated haptic cues to the gastrocnemius lateralis (GL) and biceps brachii (BB) muscles. We considered these muscles, as the former is highly activated during the stance phase of walking, and the latter shows almost no significant activity and can be considered a control. We provided haptic cues during each 10% of the gait cycle (GC) and measured how accurately the participant could recognize them to analyze the relation between haptic cue recognition and muscle activation. Based on this, the first objective of the current study was to introduce a method of accurately identifying haptic cues produced using electrical stimulation (ES). The second objective was to determine if there is any association between haptic cue identification and EMG activity. Unraveling this dynamic holds immense potential for optimizing haptic technology for gait interventions, rehabilitation protocols, and other applications. By tailoring haptic feedback to individual neuromuscular responses, we can unlock the full potential of this technology to enhance mobility, independence, and safety for individuals with gait impairments.

## 2. Materials and Methods

### 2.1. Participants

Three healthy young male adults (age 29.3 ± 7.5 years; weight 71.7 ± 6.4 kg; height 175 ± 8.7 cm) were recruited through an advertisement for this study between 1 January 2024 and 24 February 2024. The inclusion criteria were no recent musculoskeletal or neuromuscular problems or injuries and no history of surgery influencing walking performance. Before participation, the participants were instructed about the study’s objectives and familiarized with the experimental protocol. The start and end of the recruitment period for this study was 1 January 2024 to 24 February 2024.

### 2.2. Equipment

**Figure 1:**
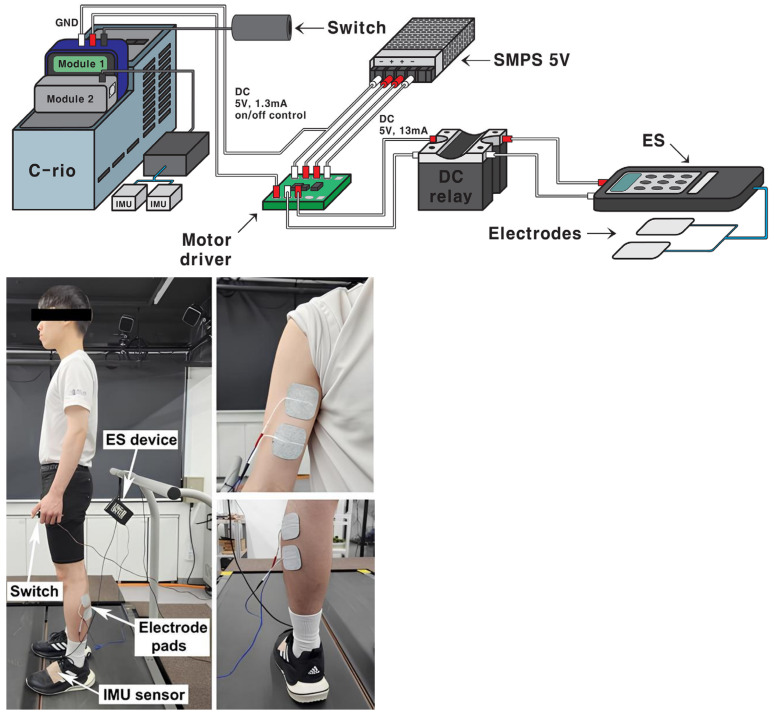
(**Top**): Instrument setup for current study. (**Bottom**): Participant holding switch while sensors are attached (**left**) and electrode pads’ locations for biceps brachii (BB) and gastrocnemius lateralis (GL) (**right**).

Figure 1 depicts the instrument setup for the current study. Our research setup was developed on a controller (cRIO-9040, National Instruments Corporation, Austin, TX, USA) to manage the workflow. The data from inertia measurement unit (IMU) sensors (MTi–630 AHRS, Xsens Technologies B.V., Enschede, The Netherlands) collected at 400 Hz were transmitted to the CAN BUS module (NI–9860, National Instruments Corporation, Austin, TX, USA) through the transceiver cable. Moreover, to deliver external control signals to the ES device (Microstim2, Sejin m.t., Seoul, Republic of Korea), a multifunction input/output module (NI–9381, National Instruments) was used. A higher current than the module could provide was required to convert the control signals into stimulation for the ES device. Therefore, a DC relay (RM1D060D20, Carlo Gavazzi, Lainate, Italy) and a motor driver (DRV8838 Single Brushed DC motor driver, Pololu Corporation, Las Vegas, NV, USA) were employed to supply the necessary current for this conversion with minimal time delay. A 3D-printed cylindrical handle with an attached switch connected to the input/output module served to deliver recognition signals.

Muscle activation envelopes were obtained using EMG sensors (Trigno™ Mini, Delsys, Inc., Natick, MA, USA) which were synchronized using a trigger module (Trigger Module, Delsys, Inc., Natick, MA, USA) connected to the input/output module.

**Figure 2:**
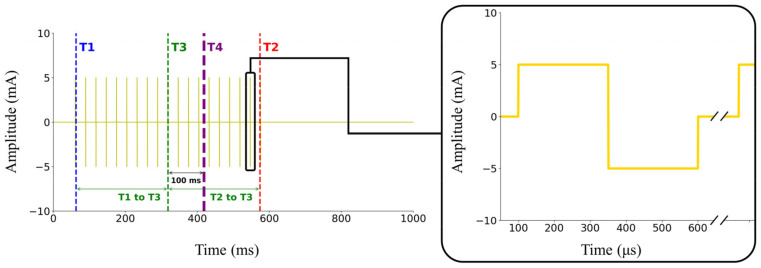
Left: Electrical stimulation (ES) output after controller’s command as conceptual illustration. Right: Example of single pulse used during ES, characterized by biphasic waveform.

During the control of the controller by the cRIO system, we observed a delayed response from the electrical stimulus of the ES device, illustrated in Figure 2, where T1 is the stimulus-on timing and T2 is the stimulus-off timing. The stimulus lasted for 10% of the GC plus 411 ms, totaling 517.7 ms, considering that this study’s average walking stride duration was 1066.8 ms. Previous research [14] reported a delay of 0.1 s between the stimulus and the force generated in the muscle by ES. Therefore, we shifted the midpoint of the electrical stimulus (T3) back by 0.1 s to account for the muscle’s force generation delay. Since our controller was configured for a 1 s duration for each GC, the average error observed among subjects was −2.26 ± 1.39%. Given that this result was analyzed using discrete intervals of 10%, the observed errors appear reasonable. Finally, we aligned the midpoint of the delayed response from the electrical stimulus (T4) to the GC percentage, which we determined as the target for ES.

### 2.3. Experimental Procedures

#### 2.3.1. EMG Data Collection

Surface EMG data on the left-side GL and right-side BB muscles during normal walking were recorded using a Delsys wireless EMG system (Trigno^TM^ Wireless System, Delsys Inc., Natick, MA, USA) synchronized with IMU at the sampling frequency of 4000 Hz. We collected EMG data in a separate walking trial so that the ES pulse would not interfere with the EMG signals. Moreover, we collected data on the right-side BB, as the participants used the left hand to push the switch. We used normal walking data with the same speed from a reference measurement to estimate the right-side heel strike (HS) based on the left side, assuming identical stride lengths for both sides.

Before attaching the EMG electrodes with double-sided tape, to reduce skin impedance and improve connectivity, the skin was carefully prepared by shaving the hair, rubbing the location with an alcohol swab, and letting it air dry. Each EMG electrode was carefully secured using hypoallergenic tape to prevent skin artifacts during walking. The EMG electrodes’ locations were verified by performing specific muscular contractions according to the SENIAM guidelines [15]. An experienced biomechanist always placed the EMG sensors to control for differences in preparation and placement technique.

Before normal walking trials, to identify the maximum voluntary contraction (MVC) of the studied muscles, the participants contracted the muscle with the greatest voluntary effort against the resistance applied manually at the end of the limb. Afterward, participants walked for 5 min to record the EMG data during normal walking. All walking trials were performed at 1.25 m/s (4.5 km/h) on a treadmill with no incline.

#### 2.3.2. Threshold Identification

**Figure 3:**
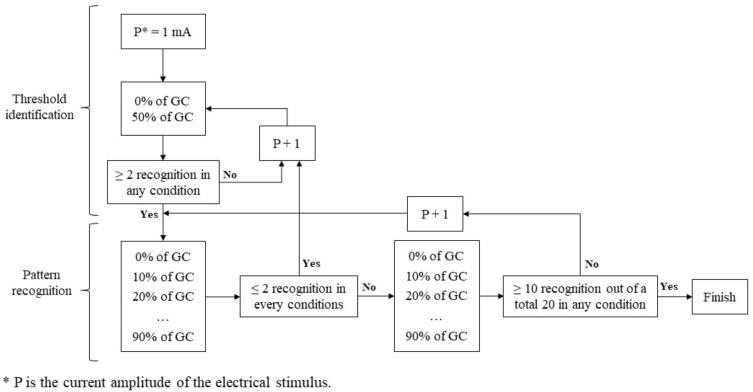
Diagram of threshold identification and pattern recognition.

A previous study [16] showed that applying the same amplitude to all participants is ineffective. Therefore, we adjusted the current amplitude individually to ensure the stimulus was neither strong (leading to recognition) nor weak (leading to failure to perceive the stimulus). This study defined the threshold as the minimum power amplitude of ES that met our criteria. Before applying the detailed intervals, we conducted a threshold identification process to quickly identify an approximate power amplitude. This preliminary step was essential to rapidly establish a baseline for more precise adjustments tailored to each participant and to prevent the experiment from becoming excessively time-consuming. Figure 3 shows the diagram of the threshold identification and following pattern recognition process.

To identify this threshold, we conducted a series of walking trials where participants received ES at specific moments with a particular power amplitude (mA). Participants walked for about 1 min on a treadmill at a speed of 1.25 m/s, with IMU sensors and ES pads attached to the target muscle. Each participant walked 10 gait cycles and received 10 electrical stimuli at designated moments, with intervals between stimuli randomized between three and six gait cycles to reduce predictability. Recognition was considered successful if the participant activated the switch within 2 s of the haptic cue. This 2 s interval was selected to ensure adequate time for the participant to determine whether the stimulus had been perceived, given that the interval between consecutive stimuli exceeded 2 s.

The power amplitude and stimuli timing in units of GC were used as independent variables, while the pulse width and frequency of electrical stimuli were fixed at 250 µs and 35 Hz, respectively. A biphasic rectangular pulse was used to shape the stimulus. Following the previous study [16], we gradually increased the current from a lower amplitude to identify the appropriate level. We initially tested at 0% and 50% of the GC, starting from 1 mA. If there were fewer than two recognitions at both timings, we increased the current by 1 mA and repeated the process. If there were two or more recognitions at either 0% or 50% GC, which we set as the criteria, we proceeded with a more detailed grid search to find the pattern recognition based on GC using the identified threshold.

#### 2.3.3. Pattern Recognition

After determining the ES threshold current magnitude, the participant received 10 electrical stimuli at each 10% interval of the GC, starting from 0% and ending at 90%. If the participant identified two or fewer stimuli in all 10 GC intervals on the first 10 trials, we increased the stimulus current magnitude by 1 mA and repeated the threshold identification process until this criterion was met. To minimize the effect of human error, we conducted a second round of pattern recognition. If the sum of successful recognitions of all 10% GC intervals was lower than 50% (10 out of 20), we increased the power amplitude by 1 mA and started the pattern recognition again. Once the sum of successful recognitions of any 10% GC intervals was 50% or higher (10 out of 20), we set that current level as the threshold and concluded the experiment. We included a 5 min rest before increasing the power amplitude for the following pattern recognition process to prevent muscle fatigue from repeated ES.

### 2.4. Data Analysis

In this study, an IMU-based gait detection algorithm was designed similarly to a previous study [17]. The GC percentage was estimated using the sagittal plane angular velocity of the IMU sensor attached to the top of the left foot. We set the zero-crossing point in the negative direction as a HS. The current GC percentage was obtained by dividing the time increment from the previous detected point by the stride time. The stride time was estimated using the average of the last three measured strides. A real-time low-pass filter (cut-off frequency at 6 Hz, first-order) was applied to smooth out the IMU data for the algorithm.

A fourth-order Butterworth band-pass filter with 50 and 450 Hz cut-off frequencies was used to filter EMG data. To reduce the noise, a fourth-order zero-phase lag Butterworth low-pass filter with a cut-off frequency of 10 Hz was used after full-wave rectification was applied to the EMG data. Then, the EMG data for each muscle were normalized to the MVC signal. Moreover, a full GC was detected from HS to consecutive HS using IMU data. EMG data of 200 GCs were selected from the middle of the trial and normalized to 101 points and the average value was calculated.

After analyzing the EMG signals, we considered the mean of this baseline calculated over a 50 ms interval to determine where the muscle was active. A muscle is considered active when the EMG amplitude surpasses the mean baseline activity by over two standard deviations for at least 25 ms [18]. This process was supplemented by repeated visual verification as recommended [19].

To investigate the relationship between muscle activation and electro-stimulated haptic perception, we compared the values of the two datasets on a GC basis. However, perception may not align with muscle activation simultaneously, potentially leading or lagging. To resolve this issue, we utilized dynamic time warping (DTW) instead of comparing the values on a GC basis to assess the similarity between the two time-series datasets. A lower DTW distance indicates a higher similarity in the shape of two time series datasets [20]. We calculated the DTW distance between the EMG signals and recognition patterns for the two target muscles and analyzed the differences.

## 3. Results

**Figure 4:**
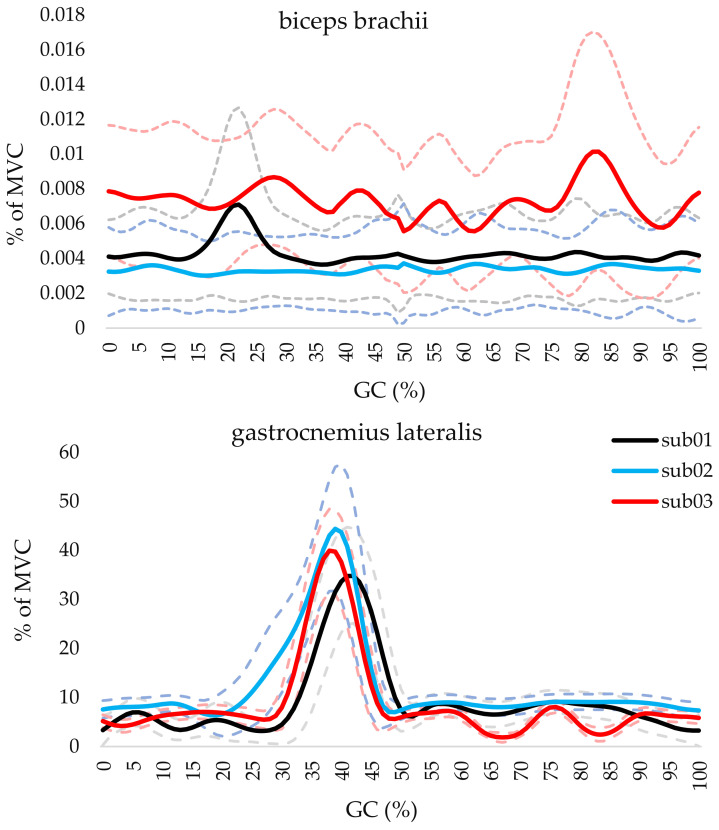
Normalized electromyography (EMG) signals (mean ± SD) of BB and GL during normal walking for three participants of current study.

Figure 4 shows the normalized EMG activity BB and GL muscles across the gait cycle for each participant. The curves represent the mean and standard deviation of the normalized muscle activity (% of maximum voluntary contraction, MVC) over the GC. For the BB muscle, the curve shows minimal activation (lower than 1% of MVC) for all participants, reflecting its control function with no significant involvement in the walking motion. In contrast, the GL muscle exhibits higher activation levels during mid-stance to pre-swing phases (approximately 25–55% of GC), indicating its role in propulsion. The muscle was identified as active during 35–55%, 26–51%, and 31–50% for sub01, sub02, and sub03, respectively.

Table 1 summarizes the electro-stimulated haptic cue threshold and pattern recogni-tion results for the BB and GL muscles. The table represents the participants’ successful recognition of haptic cues at each 10% interval of the gait cycle, ranging from 0% to 90% GC. These values indicate the frequency of correct haptic cue identifications. Bold formatting highlights intervals where participants achieved higher recognition rates equal to or greater than 50% of the total stimuli, reflecting stronger or more consistent haptic perception for these specific phases. The findings show that for the BB, a small threshold of electrical power amplitude of ES (1–5 mA) was needed for participants to feel the haptic cue. The ability to recognize patterns of haptic cues also varied across participants. Subject 1 could reliably identify the pattern during the first half of the stimulation cycles (0–50% of GC). Meanwhile, Subject 2 could recognize the pattern in some specific intervals (0–10% and 40–80% of GC). However, Subject 3 could only identify the pattern during a particular interval (10–20% of GC).

For the GL muscle, a slightly higher threshold of electrical power amplitude of ES (4–9 mA) was needed for participants to feel the haptic cue. The ability to recognize patterns was more consistent across participants for this muscle compared to the BB muscle. Subjects 1, 2, and 3 could identify the pattern in 50–70%, 40–70%, and 50–80% of the GC, respectively.

**Figure 5:**
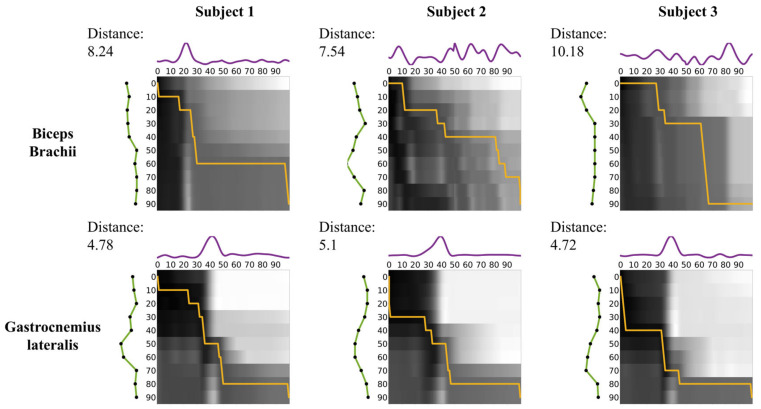
DTW distance and distance matrix between EMG signals (purple line) and recognition patterns (green line). The orange line indicates the optimal warping path derived from the DTW algorithm.

In Figure 5, the matrix in the upper row corresponds to the BB, while the matrix in the lower row corresponds to the GL. Each matrix’s axes represent the GC. The intensity in the matrices reflects the Euclidean distance between EMG signals and recognition patterns, with darker shades representing smaller distances from white to black. The orange line indicates the optimal warping path derived from the DTW algorithm. The DTW distances correspond to the overall alignment costs for the BB and GL, respectively.

For Subject 1, the DTW distance between EMG and recognition in the BB was 8.24, while in the GL, it was 4.78, resulting in a relative difference of 72.3%. For Subject 2, the DTW distance in the BB was 7.54 and 5.1 in the GL, resulting in a relative difference of 47.8%. For Subject 3, the DTW distance was 10.18 in the BB and 4.72 in the GL, resulting in a relative difference of 115.8%. Across all subjects, the DTW distance in the GL was consistently lower than that in the BB. The mean DTW distance was 8.65 ± 1.36 (mean ± SD) in the BB and 4.87 ± 0.21 (mean ± SD) in the GL, resulting in a relative difference of 78.6%.

## 4. Discussion

This pilot study investigated the interplay between muscle activity and the recognition of electro-stimulated haptic cues during normal walking. Our findings provide initial data on the feasibility of using ES to deliver haptic cues and the potential influence of muscle activation on the ability to recognize these cues accurately. Our results suggest a nuanced interplay between muscle activation and haptic perception, highlighting the complexity of human neuromuscular responses to artificial sensory stimuli.

The experiment identified thresholds ranging from 1 to 9 mA for participants to perceive the ES across both muscles. These values are not comparable to those reported in prior studies investigating ES for gait rehabilitation or sensory feedback. The reason for this difference lies within the purpose of these studies. Several studies have attempted to reproduce haptic or tactile feedback using ES to activate these receptor nerves directly. These studies employed ES to stimulate the muscles, thereby generating a sensation of force and controlling the angle of the joint. This method is widely used in human interface development [21,22,23], called functional ES in the field of rehabilitation, and needs strong ES. However, in the current study, the purpose of using ES was to provide the participants with tactile cues, which felt like very gentle sensations of pressure and low-frequency vibrations.

Moreover, the amplitude of ES for tactile cues was lower for the BB than the GL across all subjects. The main reason for this finding is the thinner subcutaneous fat layer around the upper arm area compared to the back of the leg, which made the haptic cue caused by ES more tangible for our participants.

The results partially support our hypothesis that higher muscle activation might be associated with more accurate electro-stimulated haptic cue perception. Across all three participants, the BB, a muscle minimally active during walking, exhibited a considerably variable recognition pattern for received electro-stimulated haptic cues throughout the 10% GC intervals, with a DTW distance value that was 78.6% higher than that of the GL. Conversely, the GL, a muscle actively involved in walking propulsion and highly active during the mid-stance, terminal stance, and pre-swing phases of walking, showed consistent recognition across the end of the stance phase and beginning of the swing phase of the GC for all participants. This suggests a potential link between a muscle’s inherent activity level and its ability to perceive electro-stimulation.

These findings align with previous work suggesting a link between EMG activity and haptic perception [24,25]. It seems that increased muscle activity could enhance the salience of sensory information processed in the somatosensory cortex, potentially facilitating the detection and interpretation of electro-stimulated haptic cues [26].

However, a more nuanced understanding of this relationship is necessary. While muscle activation was generally higher in the GL than the BB, there were variations in recognition accuracy across participants for both muscles. Subject 3, for instance, recognized the haptic cue during the beginning of the swing phase, where the GL is relatively inactive. This variability might be attributed to individual differences in neuromuscular responses or slight variations in electrode placement. Moreover, this suggests that factors beyond muscle activation, potentially including individual differences in sensory perception or cognitive processing, may also play a significant role.

In response to the observed individual variability in neuromuscular response patterns, we propose an adaptive approach to manage and accommodate these differences in future research. This method involves a dynamic calibration of ES parameters, such as threshold, personalized for each participant to account for variations in sensitivity and muscle activation thresholds. This individualized calibration aims to enhance the consistency of haptic cue perception by adjusting to each person’s unique neuromuscular profile. Furthermore, integrating additional physiological measures, such as real-time feedback on EMG signal variability and cognitive load indicators, could refine this approach, offering a more comprehensive understanding of how neuromuscular and sensory systems interact with ES during walking. Future studies could apply machine learning algorithms to analyze these physiological data in real-time, enabling the development of a closed-loop system that continuously adapts stimulation parameters based on the user’s ongoing response patterns.

This pilot study serves as a foundation for future investigations into the interplay between muscle activity and electro-stimulated haptic perception during walking. Several limitations are acknowledged. These results should be interpreted with caution due to the small sample size. In addition, inter-individual variations in peripheral neural innervation patterns or somatosensory processing could influence perception. Future studies should include a larger and more diverse participant pool to enhance the generalizability of the results.

Additionally, the study design employed relatively simple haptic cues, which have constant amplitude pulse stimuli. Future research could explore more complex haptic patterns and investigate how they influence recognition accuracy and their potential for conveying specific information during walking. Furthermore, the current study focused solely on muscle activity as a measure of neuromuscular engagement. Future investigations could incorporate additional physiological measures to provide a more comprehensive understanding of the central nervous system processes underlying haptic perception during walking.

Moreover, the chosen muscles (BB and GL) might not comprehensively represent the complex relationship between muscle activity and haptic perception. Future studies should explore a broader range of muscles with varying activity levels during gait.

Finally, we used a sample of three participants in this pilot study, as our primary objective was to investigate the feasibility of using electro-stimulated haptic cues for recognizing muscle activity patterns during walking. In this pilot study, statistical significance testing was not conducted due to the limited sample size and the exploratory nature of the research. The small sample size allowed us to explore individual variations and refine our methods without extensive resource use. This approach is consistent with pilot study methodologies, which aim to identify preliminary trends and inform sample size calculations for future studies. Future research will involve larger samples to provide statistical power and generalizability.

## 5. Conclusions

In conclusion, this pilot study provides preliminary evidence for a potential association between muscle activity and electro-stimulated haptic pattern recognition during normal walking. ES delivered haptic cues, and muscle activation potentially influenced perception accuracy. The GL muscle, actively engaged in walking, showed a more consistent recognition pattern compared to the less active BB. These findings support a link between muscle activity and haptic perception. However, individual differences signify the need to explore optimal stimulation parameters and haptic cue design further. Future research with larger participant pools, incorporating more complex haptic patterns and utilizing additional physiological measures, is warranted to elucidate the intricate interplay between muscle activity and haptic perception and their potential applications in gait interventions, rehabilitation protocols, and assistive technologies.

## Figures and Tables

**Table 1:** Electro-stimulated haptic cue threshold and pattern recognition results for BB and GL muscles across each 10% interval of gait cycle (GC) and dynamic time warping (DTW) distance between EMG signals and recognition patterns for three participants in the current study. Bold formatting highlights intervals where participants achieved higher recognition rates equal to or greater than 50% of the total stimuli.

			GC %										
**Muscle**	Participant	Threshold (mA)	0	10	20	30	40	50	60	70	80	90	DTW Distance
**BB**	sub01	5	**18**	**14**	**17**	**16**	**14**	2	5	2	2	4	8.24
	sub02	3	**12**	9	7	2	**10**	**13**	**19**	**12**	3	6	7.54
	sub03	1	6	**10**	6	0	0	0	0	0	1	2	10.18
**GL**	sub01	5	3	2	0	5	4	**12**	**10**	0	1	0	4.78
	sub02	9	5	1	1	4	**11**	**15**	**15**	8	2	0	5.1
	sub03	4	8	0	2	0	4	**13**	**17**	**19**	3	2	4.72

## Data Availability

The dataset generated and analyzed during the current study is available from the corresponding author upon reasonable request.
